# Effects of water avoidance stress on peripheral and central responses during bladder filling in the rat: A multidisciplinary approach to the study of urologic chronic pelvic pain syndrome (MAPP) research network study

**DOI:** 10.1371/journal.pone.0182976

**Published:** 2017-09-08

**Authors:** Zhuo Wang, Harriet H. Chang, Yunliang Gao, Rong Zhang, Yumei Guo, Daniel P. Holschneider, Larissa V. Rodriguez

**Affiliations:** 1 Department of Psychiatry and Behavioral Sciences at the University of Southern California, Los Angeles, California, United States of America; 2 Department of Urology at the University of Southern California, Los Angeles, California, United States of America; University of California Los Angeles, UNITED STATES

## Abstract

Stress plays a role in the exacerbation and possibly the development of functional lower urinary tract disorders. Chronic water avoidance stress (WAS) in rodents is a model with high construct and face validity to bladder hypersensitive syndromes, such as interstitial cystitis/bladder pain syndrome (IC/BPS), characterized by urinary frequency and bladder hyperalgesia and heightened stress responsiveness. Given the overlap of the brain circuits involved in stress, anxiety, and micturition, we evaluated the effects chronic stress has on bladder function, as well as its effects on regional brain activation during bladder filling. Female Wistar-Kyoto rats were exposed to WAS (10 days) or sham paradigms. One day thereafter, cystometrograms were obtained during titrated bladder dilation, with visceromotor responses (VMR) recorded simultaneously. Cerebral perfusion was assessed during passive bladder distension (20-cmH_2_O) following intravenous administration of [^14^C]-iodoantipyrine. Regional cerebral blood flow was quantified by autoradiography and analyzed in 3-dimensionally reconstructed brains with statistical parametric mapping. WAS animals compared to controls demonstrated a decreased pressure threshold and visceromotor threshold triggering the voiding phase. At 20-cmH_2_O, VMR was significantly greater in WAS animals compared to controls. WAS animals showed greater activation in cortical regions of the central micturition circuit, including the posterior cingulate, anterior retrosplenial, somatosensory, posterior insula, orbital, and anterior secondary (“supplementary”) motor cortices, as well as in the thalamus, anterior hypothalamus, parabrachial and Barrington nuclei, and striatum. Seed analysis showed increased functional connectivity of WAS compared to control animals of the posterior cingulate cortex to the pontine parabrachial nucleus; of the Barrington nucleus to the anterior dorsal midline and ventrobasilar thalamus and somatosensory and retrosplenial cortices; and of the posterior insula to anterior secondary motor cortex. Our findings show a visceral hypersensitivity during bladder filling in WAS animals, as well as increased engagement of portions of the micturition circuit responsive to urgency, viscerosensory perception and its relay to motor regions coordinating imminent bladder contraction. Results are consistent with recent findings in patients with interstitial cystitis, suggesting that WAS may serve as an animal model to elucidate the mechanisms leading to viscerosensitive brain phenotypes in humans with IC/BPS.

## Introduction

Chronic emotional stress plays a role in the exacerbation and possibly the development of functional lower urinary tract disorders (LUTD) [1–5]. These disorders can be viewed as a spectrum of bladder hypersensitivity syndromes sharing the common symptoms of urinary frequency and urgency, with bladder pain syndrome/interstitial cystitis (IC/BPS) patients experiencing pain as an additional symptom. Work over the past decade in chronic pain disorders, including IC/BPS, irritable bowel syndrome, fibromyalgia and vulvodynia, suggests the presence in these conditions of altered sensory perception [6–8]. Specific to IC/BPS, recent work has shown that patients compared to healthy controls show increased functional brain activation in the full bladder state broadly across sites in brain circuits serving sensory perception and pain [9]. The possibility of altered central processing of sensory input is paralleled by epidemiologic studies documenting that a substantial number of IC/BPS patients report stress-related symptom onset and/or severity, difficulty coping with stressful situations, and symptoms of depression, anxiety, and panic disorder [5,10–15]. However, the relationship between chronic stress, bladder function and brain functional responses remains unclear. The current study addresses this in an animal model by prospectively examining the relationship between chronic stress exposure, bladder function, hyperalgesia and functional brain activation. Our prior work has shown that chronic psychological stress in high-anxiety rats induces urinary frequency, sustained bladder hyperalgesia, tactile hindpaw allodynia and suprapubic hyperalgesia [16,17]. Using this animal model, we now examine the effects chronic psychological stress has on functional bladder dynamics, as well as functional brain responses during bladder filling. We hypothesize that rats exposed to water avoidance stress compared to non-stressed controls will during passive bladder filling show abnormal bladder function and sensitivity, as well as increased activation of brain regions within the micturition circuit.

## Materials and methods

### Animals

Adult, female Wistar-Kyoto rats (WKY strain 008) were purchased from a commercial vendor (Charles River, Wilmington, MA, USA). Rats were group housed under standard vivaria conditions (lights off from 7 p.m. to 7 a.m.). WAS and control groups were separately housed in groups of two with direct bedding (7090 Teklad sani-chips, Envigo), and ad libitum access to standard lab chow (Laboratory Rodent Diet 5001, Constant Nutrition^®^) and water (internal reverse osmosis systems).

Experimental procedures were approved by the University of Southern California’s Institutional Animal Care and Use Committee (protocol #20263) and were conducted in accordance with the National Research Council Guide for the Care and Use of Laboratory Animals [18]. Fourteen rats were randomized to 10-day water avoidance stress (WAS, n = 8, 12.3 +/- 0.4 weeks of age) or handled controls (n = 8, 13.3 +/- 0.4 weeks of age).

### Chronic psychological water avoidance stress

The current study used a validated rat model of chronic psychological stress (chronic water avoidance, WAS) which leads to bladder dysfunction characterized by frequent, small volume voids, bladder hyperalgesia, and increased nociceptive reflex responses to bladder distension [16,17,19]. A standing cube (6 x 6 x 9.5 cm^3^) was placed in the middle of the water tank (25 x 50 x 25 cm^3^). The water was filled up to 2 cm away from the top. The WAS rat was placed on top of the cube with the water at room temperature for 1 hour every day for 10 consecutive days. The control rat was handled and kept in its housing cage in the experimental room for 1 hour.

### Surgical procedures

On day 11, under isoflurane anesthesia (1–2% in the gas mixture of 30% oxygen and 70% nitrogen) animals received a percutaneous venous catheter, placed in the right external jugular vein. α-chloralose (40 mg/ml, Sigma Aldrich, St. Louis, MO, USA) was dissolved in 20% β-cyclodextrin (MW 1380, Sigma Aldrich) and administered intravascularly as a bolus at 40 mg/kg. The isoflurane anesthesia was maintained at 1% for 30 minutes to allow the α-chloralose to take effect. To obtain the visceromotor response (VMR), two fine and insulated silver wire electrodes (0.05 mm diameter, A-M Systems, Everett, WA, USA) with exposed tips were embedded into the left abdominal external oblique muscle (EOM). The sampling rate was set to 1kHz for both electromyography (EMG) channels. A PE-50 catheter was inserted into the bladder through the urethra outlet. The bladder catheter was connected to a 3-way connector with a syringe pump and a pressure sensor to infuse normal saline and obtain a cystometrogram (CMG).

After all surgical procedures were completed, animals received an infusion of α-chloralose (15 mg/kg/hr), sufficient to provide continuous conscious sedation to the animals. We chose light sedation using α-chloralose because of its favorable profile in maintaining cerebral function intact [20] as compared to isoflurane [21,22] or urethane [23]. At the same time, α-chloralose when given at a low dose (15 mg/kg/hr) allows assessment of bladder urodynamics and the micturition reflex in rats [24].

### Measurement of CMG and VMR recordings

CMG and VMR were obtained 1 hour after discontinuation of isoflurane anesthesia with animals maintained on a heating pad (37.0°C) under light sedation with α-chloralose (15 mg/kg/hr, i.v.). The bladder was infused with normal saline (0.9%, 0.1 mL/min) at room temperature with an open urethral outlet, thus allowing the animal to void. CMG and VMR were recorded by a data acquisition system (MP150; Biopac Systems Inc., Goleta, CA, USA) and stored on a computer for data analysis. CMG and VMR were also obtained during isotonic bladder distention (0 → 10 → 20 → 30 → 40 cmH_2_O) with urethral occlusion.

During the continuous bladder infusion, the latency of the first void/leak (LFV) was measured as the latency from the beginning of bladder filling to occurrence of the first void/leak. The bladder capacity (mL) was calculated as LFV (min) × infusion rate (0.1 mL/min). The maximum intravesical pressure (IVPmax), and pressure threshold to first void/leak (PT) were obtained. These parameters were calculated on first void to ensure the bladder was completely emptied (not carrying a residual volume) at the beginning of the continuous bladder infusion and CMG measurements. The bladder pressure evoked VMR was referred to as VMR threshold (cmH_2_O). The normalized ratio of VMR threshold/IVPmax was calculated for each animal. During bladder distension, a 25-second duration of VMR area under curve (AUC) was measured at each level of bladder pressure from 0 to 40 cmH_2_O.

### Functional brain mapping

Sixty minutes after completion of the VMR/CMG studies and two hours after discontinuation of the isoflurane, rats were connected via their venous catheter to a cannula extension and a mechanical infusion pump. Light sedation was maintained with α-chloralose (15 mg/kg/hr, i.v.). Rats received a final isotonic bladder distension (20 cmH_2_O). Forty-five seconds after initiating the distension, and in the absence of any voiding (confirmed by absence of a VMR), [^14^C]-iodoantipyrine (100 μCi/kg**,** Am. Radiolabelled Chem., St. Louis, MO, USA) was injected as an i.v. bolus, followed immediately by injection of the euthanasia solution (50 mg/kg pentobarbital/3M KCl). This resulted in cardiac arrest within ~5-10s and provided the temporal resolution of the brain mapping method [25].

Brains were flash frozen and serially sectioned (60 coronal 20-μm slices, 300-μm interslice distance). Autoradiographic images of brain slices along with 12 [^14^C] standards (GE Healthcare Life Sciences, Pittsburgh, PA, USA) were digitized. Regional cerebral blood flow related tissue radioactivity (rCBF) was measured by the classic [^14^C]-iodoantipyrine method [26–28]. In this method, there is a strict linear proportionality between tissue radioactivity and CBF when the data is captured within a brief interval (~10 seconds) after the tracer injection [29,30].

### Statistical analysis

#### Urodynamic studies

For CMG and VMR during bladder infusion, data is expressed as means and standard errors (means ± SE). Unpaired t-tests were performed to compare control and WAS groups. A value of P < 0.05 indicated a significant difference between groups. GraphPad Prism5 (GraphPad Software Inc., LaJolla, CA, USA) was used for the statistical analysis and graph demonstration.

#### Functional brain mapping

Image processing: Each 3-D brain was reconstructed from 57 digitized autoradiograms (voxel size: 40 x 300 x 40 μm^3^) using TurboReg [31] and a nonwarping geometric model that includes rotations and translations and nearest-neighbor interpolation. A brain template was created using our prior published methods [32], in which the background and ventricular spaces were thresholded based on their optical density. Spatial normalization consisted of a 12-parameter affine transformation followed by a nonlinear spatial normalization using 3-D discrete cosine transforms. Normalized brains were smoothed with a Gaussian kernel (FWHM = 3 x voxel dimension). Absolute amount of radiotracer of each brain was scaled to a single mean.

Analysis of regional brain activation: Statistical parametric mapping (SPM) [33] was used to identify regions showing significant rCBF changes. Significance was set at *P*<0.05 at the voxel level and an extent threshold of 100 significant, contiguous voxels to control Type I and Type II errors. In addition, we verified the SPM results with a a region-of-interest analysis. Regions of interest (ROI) were drawn manually for regions within the micturition circuit. Regions were identified by a rat brain atlas [34]. A functional ROI was created by combining the anatomical ROI with its significant SPM cluster by logical conjunction. Mean optical density was extracted for the ROI using the Marsbar toolbox [35]. The directionality of rCBF changes was analyzed with Student’s t-tests (P<0.05).

Seed correlation analysis: Functional ROIs as defined above for the right posterior cingulate cortex, the right Barrington nucleus, as well as the right posterior insula underwent a seed correlation analysis. Seed correlation was visualized in SPM across the whole brain, with color-coded correlation coefficients (*P* < 0.05, extent threshold > 100 significant, contiguous voxels).

Additional supporting information is also available through the Open Science Framework (OSF; http://osf.io), a data sharing network.

## Results

### CMG and VMR recordings

**Fig 1:**
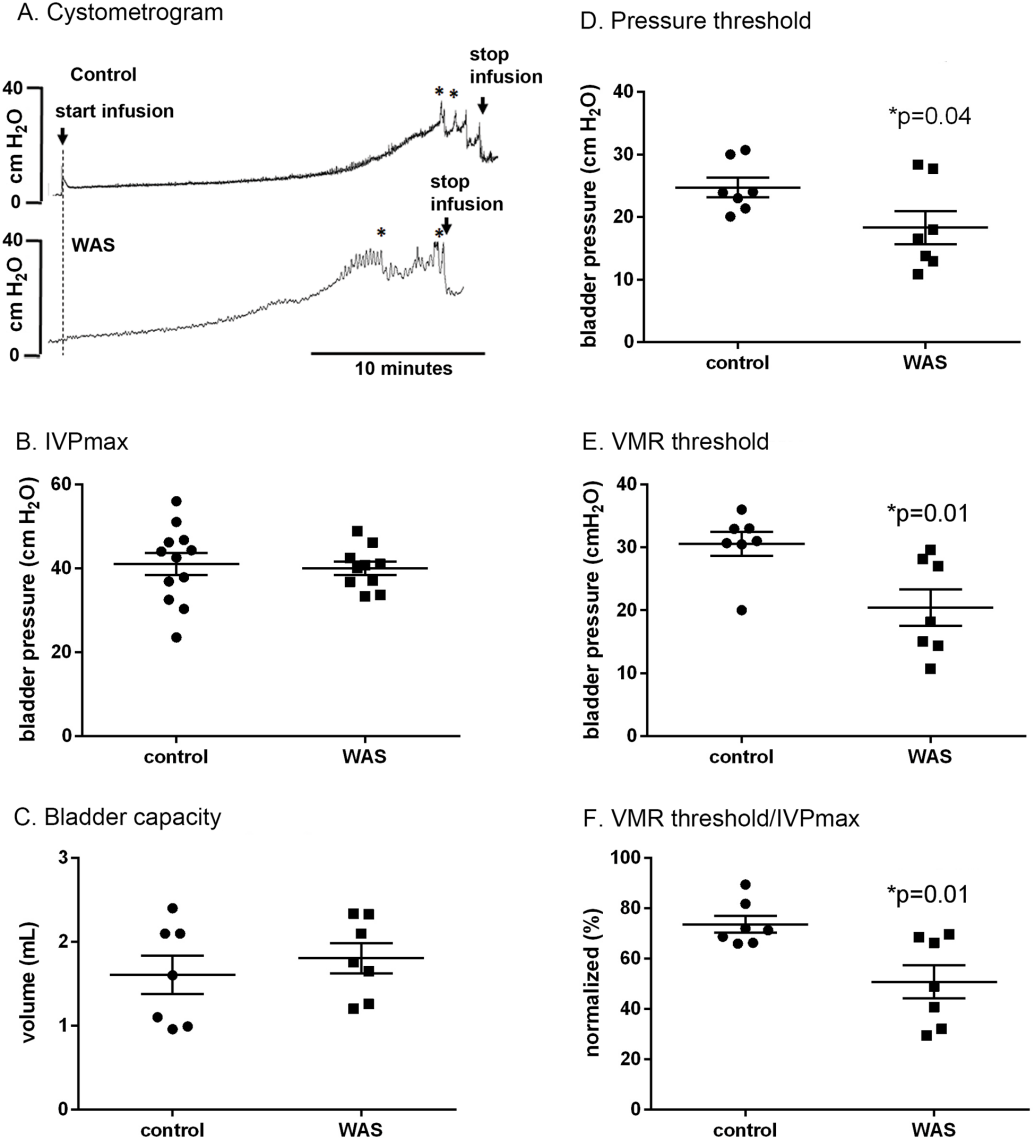
Statistical analysis of cystometrogram and visceromotor reflex (VMR +/- standard error) recordings during bladder infusion. Shown are the tracings of (A) cystometrogram, (B) IVPmax (maximum intravesical pressure), (C) bladder capacity, (D) pressure threshold to first void/leak, (E) VMR threshold, and (F) VMR threshold/IVPmax for WAS and control animals. * indicated when voiding happened. The bar on the vertical axis of Figure (A) is a calibration bar, whose length represents a bladder pressure of 40 cmH_2_O.

During voiding, WAS animals showed a significant decreased PT triggering the voiding phase (P = 0.04, Fig 1D). The VMR evoked by bladder distension in WAS animals appeared at a lower IVP during bladder filling compared to controls (P = 0.01, Fig 1E). The percentage of VMR threshold pressure/IVPmax also showed the VMR to appear significantly earlier in WAS animals compared to controls (P = 0.01, Fig 1F). All these are surrogates for bladder hypersensitivity. The maximum IVP and bladder capacity showed no significant changes between control and WAS groups.

**Fig 2:**
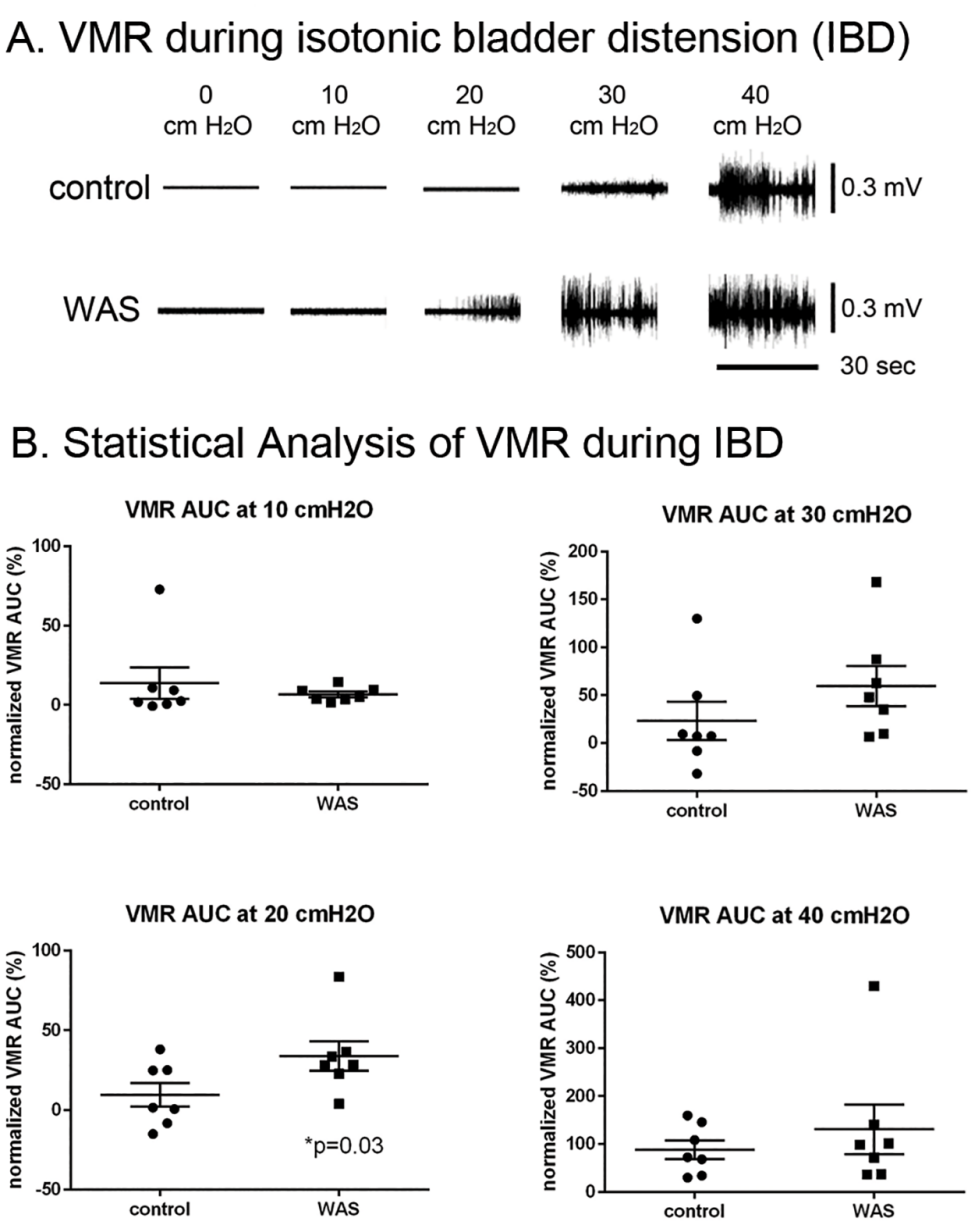
**(A) Representative examples of visceromotor reflex (VMR)** in the control and WAS animals at each level of bladder pressure between 0 and 40 cmH_2_O. **(B) During isotonic bladder distension, VMR AUC (VMR +/- standard error) in WAS significantly increased compared to controls** at 20 cmH_2_O (P = 0.03). VMR: visceromotor reflex. AUC: area under the curve. Note different scales used at different pressure levels. WAS: water avoidance stress.

During isotonic bladder distension, VMR AUC was measured from both experimental groups (Fig 2A). At 0 cmH_2_O, both control and WAS groups did not have the appearance of VMR. When the isotonic pressure reached 10 cmH_2_O, few VMRs were evoked in both groups. At 20 cmH_2_O, VMR AUC significantly increased in WAS animals compared to controls (P = 0.03, Fig 2B). At 30 cmH_2_O the VMR AUC remained increased in WAS compared to control animals, with no group difference (ceiling effect) noted at 40 cmH_2_O. In summary, WAS rats compared to control rats showed lower VMR thresholds and pressure thresholds, and greater VMR responses to passive bladder distension.

### Functional brain mapping

**Fig 3:**
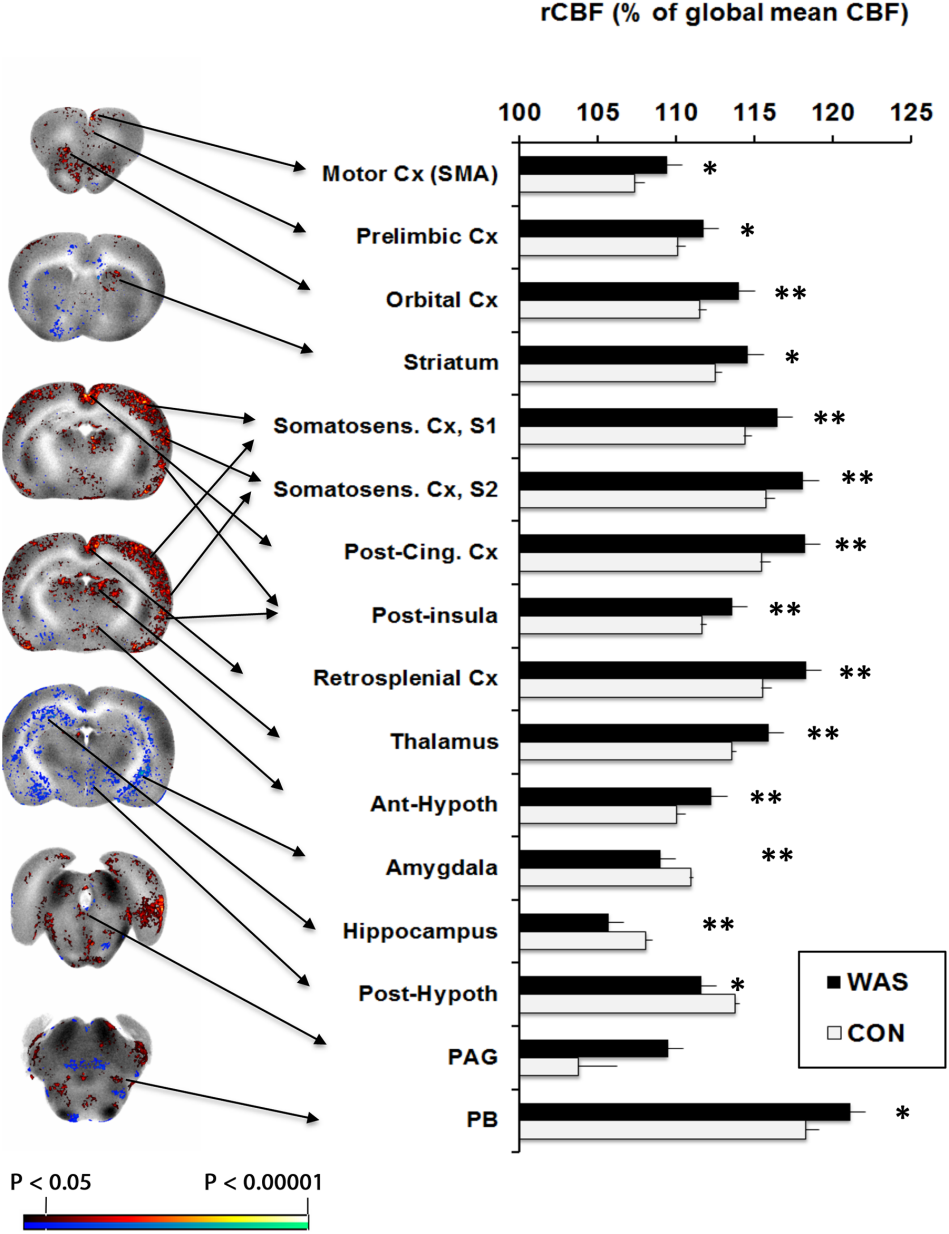
Differences in functional brain activation during bladder filling of WAS rats compared to controls. **Left column:** Statistical parametric maps showing significant difference (P < 0.05 for clusters of greater than 100 contiguous significant voxels) in brain activation in sedated WAS rats compared to controls are superimposed on gray-scale coronal sections showing the rCBF distribution of the template brain. **Right column:** ROI analysis shows group mean rCBF (± S.E.) for select brain regions in the micturition circuit, in which rCBF in each animal was normalized by its whole brain mean perfusion. * P < 0.05, **P < 0.005 for clusters of greater than 100 significant, contiguous voxels. Abbreviations: Cx (cortex), PAG (periaqueductal gray), PB (parabrachial nucleus).

**Fig 4:**
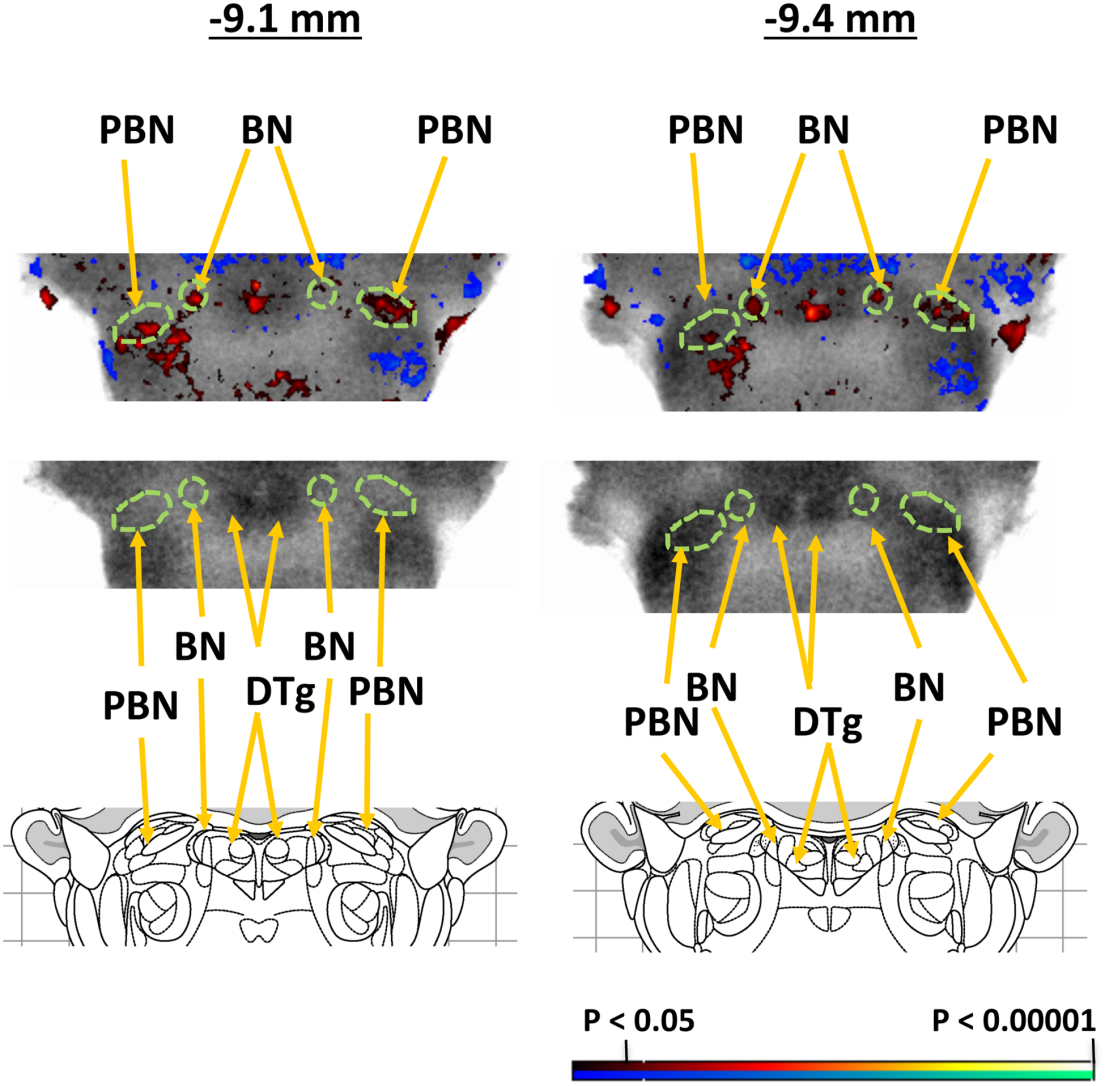
Detailed view of functional brain activation during bladder filling of WAS rats compared to controls at the level of the pontine micturition center (9.1 and 9.4 mm posterior to bregma). Significant differences in regional CBF during bladder filling of WAS rats compared to controls (red indicates increased rCBF, blue indicates decreased rCBF). Also shown are the region-of-interest definition (green) for the Barrington nucleus (BN) and parabrachial nucleus (PBN) used in our analyses, as well as the landmark for the dorsal tegmentum (DTg).

**Fig 5:**
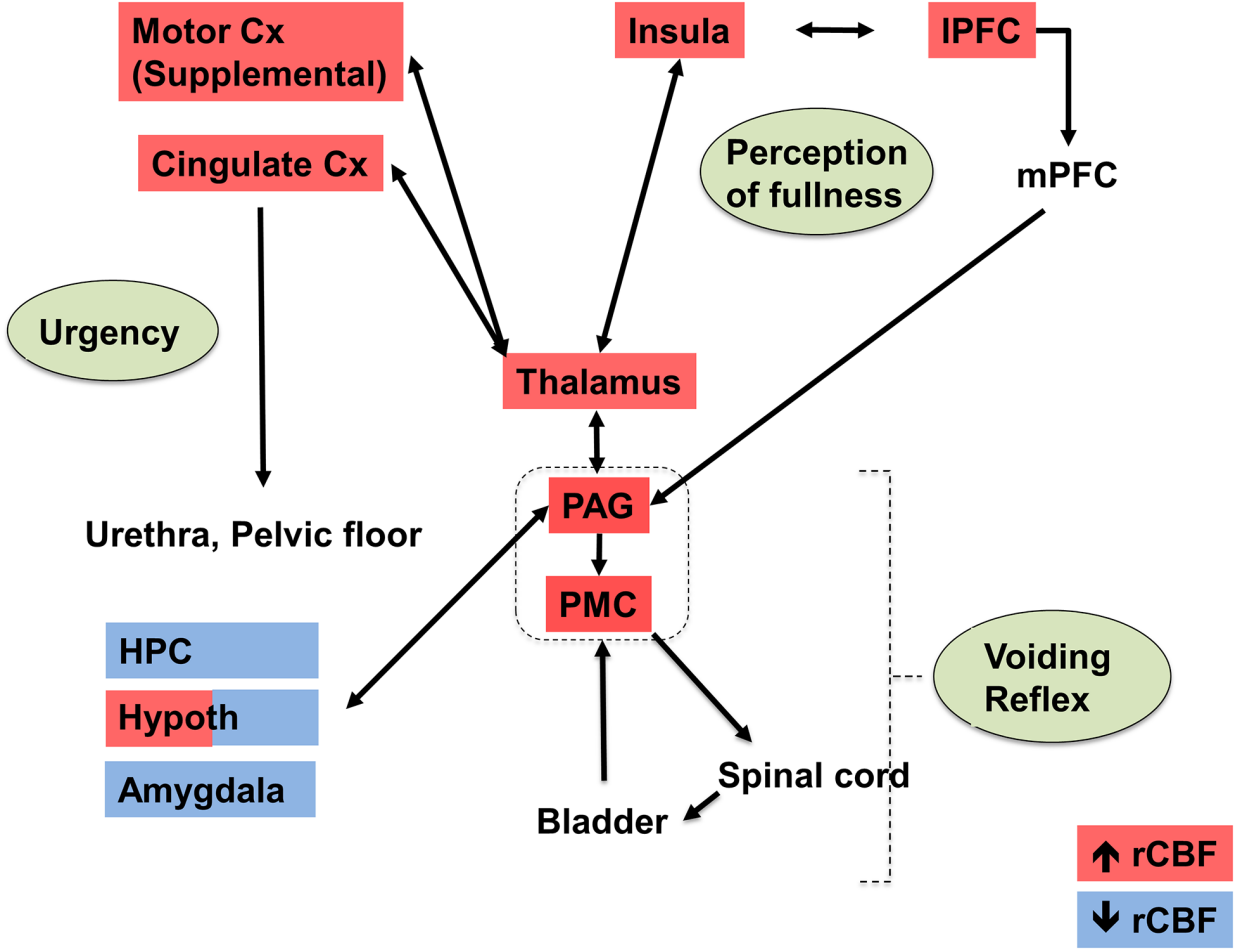
Summary of the significant changes in regional cerebral blood flow (rCBF) of WAS vs. control rats during passive bladder filling as related to a simplified model of the micturition circuit. Data are summarized from the SPM analysis shown in Fig 3, with red indicating significant increases in rCBF and blue indicating significant decreases in rCBF. Abbreviations: Cx (cortex), HPC (hippocampus), lPFC (lateral prefrontal cortex), mPFC (medial prefrontal cortex), PAG (periaqueductal gray), PMC (pontine micturition center, parabrachial/Barrington nucleus complex), rCBF (regional cerebral blood flow). Adapted from Griffiths [36] and DeGroat and Yoshimura [37].

Functional brain activation during moderate bladder distension (20 cmH_2_O) showed significantly greater rCBF (P<0.05, min. cluster size 100 voxels) in WAS compared to control rats, most broadly in posterior cingulate, anterior retrosplenial, somatosensory (primary, secondary) and posterior insular cortices (right > left), and the anterior thalamus (anterior ventral, lateral dorsal, mediodorsal, paraventricular) and lateral habenula. Significant increases in rCBF were also noted in anterior, secondary motor cortex, medial prefrontal (orbital, prelimbic) and caudomedial entorhinal cortices, as well as the dorsomedial striatum, anterior hypothalamus, superior colliculus, median raphe, and parabrachial/Barrington nucleus complex. Significant decreases in rCBF were noted in the dorsal hippocampus, posterior hypothalamus, amygdala and cerebellar cortex (Fig 3). SPM results were confirmed by ROI analysis. Fig 4 shows additional detail for the results of the SPM analysis and definition of the ROIs at the level of the pons. More specifically ROI analysis of Barrington’s nucleus and the parabrachial nucleus showed significant group differences (P<0.05, min. cluster size 100 voxels) when ROIs were examined individually or when they were pooled. Significant changes in rCBF as they relate to the micturition circuit are summarized in Fig 5.

**Fig 6:**
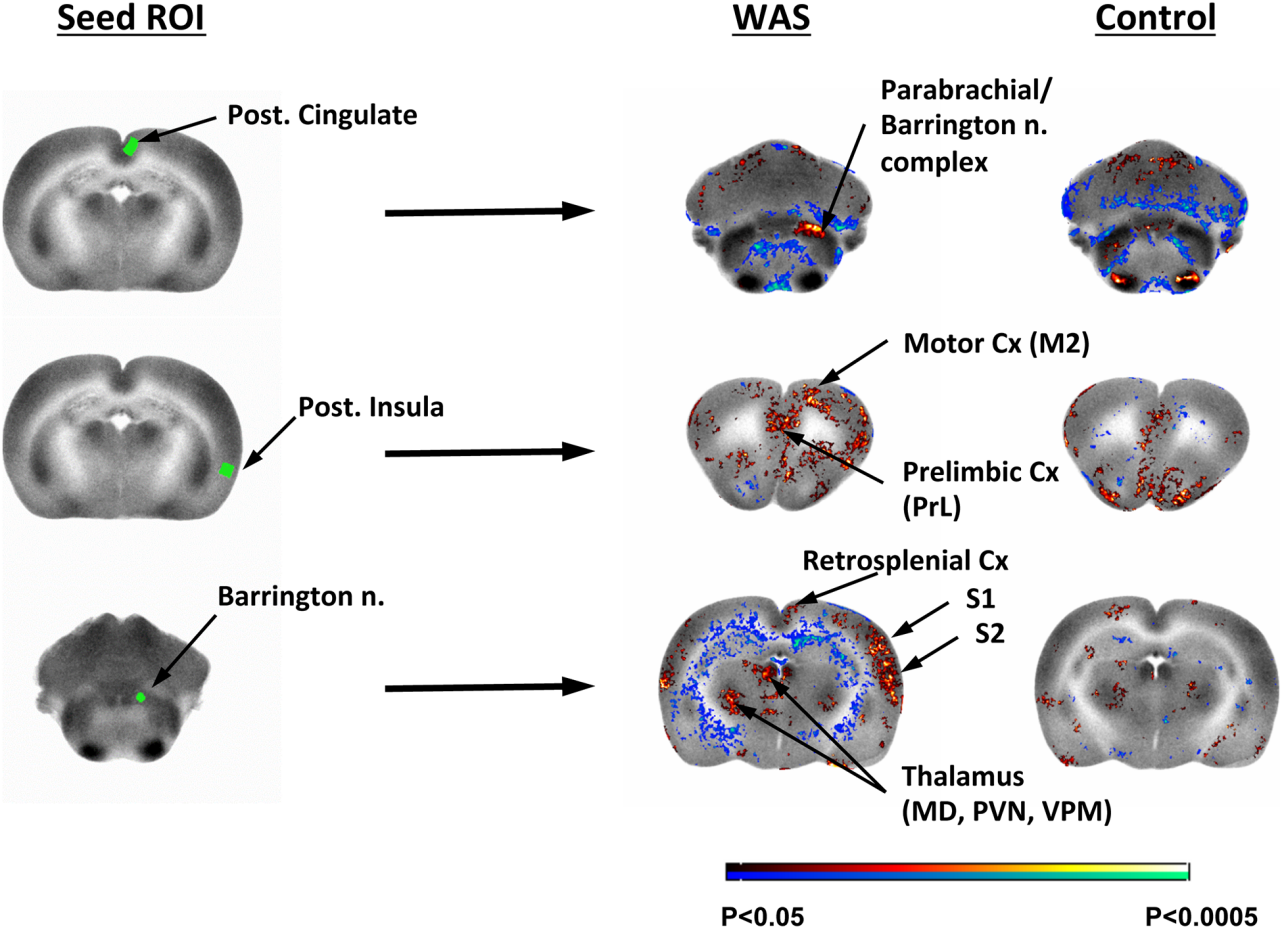
Seed correlation of functional activity across the whole brain during bladder filling. WAS compared to control rats show greater functional connectivity of **(row 1)** the posterior cingulate to the parabrachial/Barrington nucleus complex, **(row 2)** the posterior insula to anterior secondary motor cortex (M2), and **(row 3)** the Barrington nucleus to the anterior, dorsal midline thalamus (mediodorsal, MD; paraventricular, PVN), as well as the ventrobasilar thalamic complex, primary and secondary somatosensory cortex (S1, S2) and retrosplenial cortex.

Because of the broad and significant group difference noted in the posterior cingulate, this region was used in a seed correlation analysis. This showed significant positive correlation (P<0.005, min. cluster size 100 voxels) with the parabrachial nucleus in WAS animals but not in controls (Fig 6). Seed correlation of the right Barrington nucleus showed a significant positive correlation with midline cortex (mid/posterior cingulate, anterior retrosplenial), as well as somatosensory cortex (primary, secondary) and anterior secondary motor cortex in WAS animals but not in controls. Subcortically WAS rats, but not control animals showed a significant positive functional connectivity to the anterior, dorsal midline thalamus (mediodorsal, paraventricular), ventrobasilar thalamic complex, lateral habenula, dorsolateral striatum, and rostral periaqueductal gray. A significant negative correlation was noted with the posterior insula in WAS rats but not in controls. Seed correlation of the posterior insula showed significant positive correlations with anterior, secondary motor cortex (M2) in WAS animals but not in controls (Fig 6).

## Discussion

In 2008, the National Institute of Diabetes and Digestive and Kidney Diseases (NIDDK) initiated the Multi-disciplinary Approach to the Study of Chronic Pelvic Pain (MAPP) Research Network to rigorously study urological chronic pelvic pain syndrome (UCPPS) using an interdisciplinary approach [38]. UCPPS include IC/BPS and chronic prostatitis/chronic pelvic pain syndrome (CP/CPPS). Part of the MAPP network has focused on the validation of rodent models that reflect multiple key characteristics of human UCPPS and provide enhanced clinical significance to mechanistic studies. Strategies for evaluating animal models of UCPPS were developed [39]. The WAS model is one such model which reflects the stress variability seen in the human conditions and manifests the key features of urinary frequency and bladder hyperalgesia experienced by patients [17].

Recent work by Deutsch et al. [9] demonstrated that female interstitial cystitis subjects compared to healthy controls show increased rCBF during bladder distension in multiple areas of the brain associated with visceral sensory perception. Significant increases were noted in the supplemental motor area (SMA), motor and sensory cortex, the insula, the hippocampus and the middle and posterior cingulate. In light of prior epidemiologic evidence suggesting a significant association of IC/BPS with stress, poor stress coping, and symptoms of anxiety and depression [5,10–15], our study’s aim was to prospectively examine in an animal model if chronic stress exposure could result in a functional reorganization of regions within the pain matrix similar to that noted in IC patients.

A rodent model of chronic psychological stress was investigated by CMG recordings of VMR, as well as the brain activity during isotonic bladder distension. WAS animals demonstrated a VMR that appeared earlier and more forcefully than in controls. A decreased PT in WAS animals indicated that the voiding appeared early, indicating bladder hypersensitivity. Because the greatest group difference in the CMG studies was observed at 20 cmH_2_O, we used this moderate physiologic stimulus during the functional brain mapping.

Bladder distension increased functional brain activation in WAS compared to control rats broadly across the supraspinal micturition circuit (Fig 5) (reviewed in [40,41]). According to this simplified model of supraspinal bladder control, afferent input from the bladder and urethra are conveyed to the periaqueductal gray (PAG) and transmitted further to the insula, where the sensations are mapped. The cingulate cortex is involved in monitoring and evaluating bladder sensations, and may control micturition via its efferent output to the PAG and, indirectly, to the pontine micturition center, including the Barrington’s nucleus and the parabrachial nucleus. The prefrontal cortex is involved in attention to bladder sensations and decisions about voiding. In addition, the SMA and the medial primary motor cortex (M1) are regions that control voluntary contraction and relaxation of the pelvic floor muscles via efferent projections to motor neurons in the sacral spinal cord. In our study, significant stress effects were noted in most of the brain regions previously noted by Tai et al. (2009) to be responsive to passive bladder distension. These authors imaging normal, anesthetized rats during bladder filling using fMRI, reported brain activation in motor cortex, cingulate and retrosplenial cortex, primary and secondary somatosensory cortex, the putamen, septum, thalamus, posterior insula, as well as the periaqueductal gray (PAG) [42]. Equivalent brain regions have also been reported to be activated in healthy volunteers [43] and in IC subjects [9] in the context of a full bladder. In addition, our analysis revealed that WAS animals compared to controls demonstrated a significant increase in rCBF in Barrington’s nucleus, as well as the adjacent parabrachial nucleus, both representing final relay regions of the micturition circuit.

One of the broadest and most significant group differences in our brain mapping was noted in the posterior cingulate/anterior retrosplenial cortex. Seed analysis for this region revealed that WAS rats compared to controls showed greater functional connectivity of the posterior cingulate to the parabrachial nucleus that functions as a relay of the supraspinal modulation of micturition [44–47]. The function of posterior cingulate cortex remains unclear at present. It is a central node in the default mode network (DMN), with widespread and heterogeneous functional connectivity across the brain, and with proposed roles in attentional control, cognition, pain perception, emotional processing, sensory integration, including bladder filling and urinary urgency [48,49]. Recent work in patients with UCPPS has shown that the posterior cingulate is functionally disconnected at rest from the DMN, and instead shows increased functional connectivity to cerebral regions that play a role in pain, sensory, motor and affective processes, including the insula, putamen, amygdala, and hippocampus [50]. Seed analysis for Barrington’s nucleus, a final relay in micturition (reviewed in [41]), showed that WAS animals compared to controls showed a significant and broader positive functional connectivity with the medial thalamus (mediodorsal, paraventricular), associated with limbic modulation [51], as well as with a ventrobasilar thalamic complex, associated with sensory integration [52,53]. Prior work has shown structural connectivity of Barrington’s nucleus to this medial thalamic region [54,55], and functional connections to a ventrobasilar thalamic region [56].

A primary brain region implicated in functional neuroimaging of chronic pain is the insula [57–59]. The insula in the rodent is currently understood to have a posterior-to-mid-to-anterior integration of interoceptive information [60]. Primary interoceptive representations of viscerosensory information are believed to be dominantly represented in the posterior insula, while the anterior insula is believed to integrate such visceral inputs with cognitive and affective aspects of subjective awareness. A role for the posterior insula has been proposed in relaying necessary sensory information to motor cortical structures for control of muscles associated with the viscera [61–63]. In our study, examining the posterior insula during bladder filling in sedated animals, WAS rats compared to controls showed greater functional connectivity of the posterior insula to rostral midline motor cortex (M2), as well as somatosensory cortex. Rostral motor cortex in the rodent has been proposed to be the equivalent of the supplementary motor area (SMA) of primates [64–66], and represents a cortical region associated with pelvic floor and gluteal muscle activation [67–71]. Our results are relevant to recent work by Kutch et al. (2015) that suggests an important role for brain motor control in disorders of chronic pelvic pain. Specifically, in male patients with chronic prostatitis/chronic pelvic pain syndrome (CP/CPPS), there are alterations in resting state functional connectivity between the supplementary motor cortex and the posterior insula [72]. Furthermore, women with interstitial cystitis/painful bladder syndrome show altered frequency distributions in intrinsic brain oscillations in viscerosensory (posterior insula), motor regions (including the medial and ventral supplementary motor areas), as well as somatosensory cortex (postcentral gyrus) [73]. Our study suggests that chronic stress may alter functional connectivity between posterior insular cortex and sensorimotor cortex.

In light of the greater prevalence and severity and symptom expression of IC/BPS in women as compared to men, the focus of our study was on female rats [74]. Past brain mapping in healthy volunteers, however, has suggested sex differences during “attempted micturition”, with increased responses in women than in men in the right thalamus and in subregions of the right prefrontal cortex [43], with others reporting greater activity of the right insula and operculum in women than in men during a filled bladder condition [75]. Such findings have been hypothesized to reflect sex differences of a ‘salience network’ [36], part of a larger ‘interoceptive network’ [43]. In anesthetized rats, visceromotor responses to bladder distention show more vigorous reflex responses in females than in males [76]. Our own work in mapping brain function during visceral pain in awake rats has also noted sex differences in the thalamus, amygdala and insula during acute colorectal distension [77]. Future studies will need to examine if such sex differences in brain responses are also noted during bladder filling in the animal model. A limitation of our study is that since we did not include a “no bladder distension” group, we cannot determine if the significant differences in rCBF noted in response to WAS are specific to the physiologic challenge of bladder filling, or if they are also apparent in the baseline condition. Collectively, our physiologic and brain mapping results suggest a hypersensitivity in WAS animals, as well as increased engagement of portions of the micturition circuit responsive to urgency and the perception of bladder fullness, including viscerosensory perception and its relay to motor regions coordinating imminent bladder contraction. A subset of the brain regions demonstrating group differences in our study are well-known to serve multiple functions. In particular, regions including the cingulate, insula, and periaqueductal gray are described not only within the micturition circuit, but also as core regions within the pain neuromatrix [78], as well as in circuits mediating fear, mood and stress [79,80]. This is consistent with our understanding of the multiple sensory and affective/motivational aspects of pain [81].
